# 25(OH) D3 alleviate liver NK cytotoxicity in acute but not in chronic fibrosis model of BALB/c mice due to modulations in vitamin D receptor

**DOI:** 10.1186/s12876-020-01248-5

**Published:** 2020-04-10

**Authors:** Ahmad Salhab, Johnny Amer, Lu Yinying, Rifaat Safadi

**Affiliations:** 1 Research center for liver diseases, Liver & Gastroenterology Units; Division of Medicine, Hadassah –Hebrew University Hospital- Jerusalem, POB12000, Ein-Kerem, Jerusalem, Israel; 2 Comprehensive Liver Cancer Center, The Fifth Medical Center of PLA General Hospital, Beijing, China

**Keywords:** Hepatic fibrosis, NK cells, Vitamin D receptor, Hypercalcemia

## Abstract

**Background:**

Low 25-Hydroxy-vitamin-D; “25(OH)-D3” serum and vitamin D receptor (VDR) levels were recently correlated to advanced fibrosis. However, VDR mechanism in liver fibrosis modulations is not well understood. In this study, we aimed to evaluate changes in liver NK cells cytotoxicity due to modulations in VDR in CCl_4_ fibrosis model following 25(OH) D3 injections.

**Methods:**

Carbon-tetrachloride (CCl_4_) hepatic-fibrosis was induced in BALB/c mice for 1 and 4 weeks as an acute and chronic fibrosis model, respectively. Along 1th to 4th weeks, vitamin D were i.p injected/2x week. Liver were assessed histologically and for proteins quantification for VDR and αSMA expressions. In vitro, potential killing of NK cells were evaluated following co-culture with primary-hepatic-stellate-cells (pHSCs) obtained from BALB/c WT-mice.

**Results:**

Systemic inflammation and hepatic-fibrosis increased along 4 weeks of CCl_4_ as indicated by serum ALT and αSMA expressions (*P* < 0.02) as well as histological assessments, respectively. These results were associated with increased NK1.1 activations and hypercalcemia. While vitamin D administrations delayed fibrosis of early stages, vitamin D worsen hepatic-fibrosis of late stages of CCl_4_. In week 4, no further activations of NK cells were seen following vitamin D injections and were associated with down-expressions of VDR (1.7 Fold, *P* < 0.004) indicating the inability of vitamin D to ameliorate hepatic fibrosis. In vitro, NK cells from the chronic model of CCl_4_ did not affect pHSCs killing and fail to reduce fibrosis.

**Conclusion:**

Vitamin D alleviate liver NK cytotoxicity in acute but not in chronic fibrosis model due to modulations in vitamin D receptor and calcium. Hypercalcemia associated with late fibrosis may inhibited VDR levels, however, may not explain the profibrogenic effects of vitamin D.

## Background

Vitamin D is an important prohormone with known effect on calcium homeostasis [1], but recently there is increasing recognition that vitamin D also is involved in cell proliferation and differentiation, it has immunomodulatory and anti-inflammatory properties [2]. The effects of vitamin D are mediated through the vitamin D receptor (VDR) [3]. VDR is a member of the nuclear receptor super-family of ligand-inducible transcription factors, which are involved in many physiological processes, including cell growth and differentiation, embryonic development and metabolic homeostasis [4]. The transcriptional activity of this receptor is modulated by several ligands, such as steroids, retinoids and other lipid soluble compounds, and by nuclear proteins acting as co-activators and co-repressors [5]. The liganded VDR heterodimerizes with the retinoid X receptor and binds to vitamin D response elements in the promoter of target genes, thereby affecting their transcription. The genomic organization of the *VDR* at locus 12q13.1 shows that the *VDR* gene itself is quite large (over 100 kb) and has an extensive promoter region capable of generating multiple tissue-specific transcripts [6].

Clinical observations have recently demonstrated that 25-OH D serum levels were significantly lower in patients with chronic hepatitis C than in controls and that low 25-OH D serum levels were associated with more severe fibrosis and lower responsiveness to interferon-based therapy in those patients [7, 8]. Other studies have shown that low 25-OH D serum levels are associated with poor liver function and more advanced stages of liver fibrosis in hepatitis C virus patients [9]. Moreover, a possible role for vitamin D in liver fibrosis has gained further support from the finding that VDR is expressed in human as well as in rat liver non-parenchymal cells, such as Hepatic stellate cells (HSCs) [10]. It has recently been suggested that VDR polymorphism is associated with primary biliary cirrhosis [11]. Vitamin D deficiency is a common phenomenon in chronic liver disease, particularly in advanced fibrosis and cirrhosis [12]. Whether this association reflects the cause of accelerated fibrosis progression or the consequence of impaired liver function in advanced disease is still unclear. In our current study, we aimed to evaluate changes in liver NK cells cytotoxicity due to modulations in VDR in CCl_4_ fibrosis model following 25(OH) D3 injections.

## Methods

### Ethics statement and animals

Male mice on the BALB/c background, 12 weeks of age, weighing 22 ± 0.5 g, received care according to NIH guidelines. Mice were purchased commercially from Harlan Laboratories, Jerusalem-Israel. All animal protocols were approved by the institutional animal care ethical committee of the Hebrew University and housed in a barrier facility under the ethic number: MD-18-154,943.

### CCl_4_ model of liver injury and fibrosis

BALB/c mice were IP injected with 0.5 ml/kg body weight CCl_4_ (1:10 v/v in corn oil from Sigma) or vehicle (corn oil) twice a week for 1 week (acute model) and 4 week (chronic model). 25(OH) D3 (Biogems, Cat# 3220632–10 mg) at the dose of 0.5 microgram/100 g body was IP injected twice a week, commencing one day after the first dose of CCl_4_. The animals were terminated 72 h. after the final CCl_4_ injection through intramuscularly anesthetize with 0.1 ml of ketamine: xylazine: acepromazine (4:1:1) per 30 g of body-weight prior to cervical dislocation. The whole livers and serum were collected for histological, cytological and biochemical analyses.

### Alanine aminotransferase (ALT)

Blood samples were collected from BALB/c male hearts at the volume of 1 ml blood; the samples were centrifuged for 5 min at 4000 rpm. Serum samples were drawn to an Eppendorf tubes, after blood centrifuge. Serum samples of 32 μl were dropped into the strip of the ALT (Reflotron Company) and analyzed by Reflovet® plus Roche.

### Liver NK cells isolation

Under deep ether anesthesia, mice were euthanized by isoflurane, USP 100% (INH), then the liver was removed and a part of the liver was transferred to Petri dish that contains 5 ml DMEM medium (Biological industries; Cat# 01–055-1A). The liver tissue was thoroughly dissected by stainless steel mesh, the cells were harvested with the medium and added to 50 ml tubes containing 10 ml DMEM, and then carefully cells were transferred to new tubes that contain Ficoll (Abcam; Cat# AB18115269). Tubes were centrifuged for 20 min, at 1600 rpm at 20 °C. The supernatant in each tube was transferred to a new tube, for another centrifuge for 10 min, at 1600 rpm at 4 °C. After the second centrifuge, the pellet in each tube was suspended in 1 ml of DMEM for NK isolation kit (StemCells; Cat# 19665)).

### Primary HSCs isolation

HSCs were isolated from 12-week-old male BALB/c mice by in situ pronase, collagenase perfusion, and single-step Histogenz gradient as previously reported (Hendriks et al., 1985; Knook et al., 1982). Isolated HSCs were cultured in Dulbecco’s modified Eagle’s medium (Mediatech) containing 20% fetal bovine serum (Hyclone) on six-well plates for 40 h. prior to end-point assays.

### Flow cytometry

Harvested mice liver NK cells were adjusted to 10^6^/ml in buffer saline containing 1% bovine albumin (Biological Industries; Cat# 02–023-5A), and were stained for the following antibodies: Anti-mice NK1.1 (murine NK cell marker) (Biogems; Cat# 83712–70), Anti CD49a (MACS; Lot# 5150716246), and anti-mice Lysosomal-associated membrane protein-1 (CD107a; NK1.1 cells cytotoxicity marker) (eBioscience; Cat# 48–1071). All antibodies were incubated for 45 min at 4 °C. The cells were washed with 0.5 ml of staining buffer and fixed with 20 ml of 2% paraformaldehyde. All stained cells analyzed with a flow cytometer (The BD LSR Fortessa™, Becton Dickinson, Immunofluorometry systems, Mountain View, CA).

### Real time PCR

#### Tissue RNA isolation

Total cellular RNA was isolated from liver tissue, using 2 ml TRI Reagent (Bio LAB; Cat# 90102331) for each cm^3^ of tissue. The samples were homogenized for 5 min at room temperature of 25 °C. Chloroform at volume of 0.2 ml (Bio LAB; Cat# 03080521) were added to each sample, incubated for 15 min at room temperature and centrifuged (1400 rpm) for 15 min at 4 °C. RNA precipitation: The supernatant in each sample was transferred to new Eppendorf, 0.5 ml of isopropanol (Bio LAB; Cat# 16260521) left for 10 min at 25 °C and centrifuged (12,000 rpm) for 10 min at 4 °C. The supernatants were removed and one ml of ethanol 75% were added to the pellet and centrifuged (7500 rpm) for 5 min. The pellets were dried in air at room temperature for 15 min, 50 μl of DEPC were added, and the samples were heated for 10 min at 55 °C.

#### cDNA preparation

Liver RNA was extracted as described above. Preparing of c-DNA was performed using High Capacity cDNA Isolation Kit (R&D; Cat # 1406197).

### Real time PCR

Real-time PCR is performed for the quantification of the expression of the gene that encoded alpha Smooth Muscle Actin **(**αSMA) and Vitamin D Receptor (VDR), compared to GAPDH as a housekeeping gene by using Taqman Fast Advanced Master Mix (Applied Biosystem; Cat # 4371130).

### Western blot analysis

Immunoblot analysis of αSMA in liver extracts performed. Following isolation, whole protein extracts were prepared in liver homogenization RIPA buffer (Sigma; R0278-50ML) with protease phosphatase inhibitor cocktail (Roche; 1,183,617,011)*.* Next, proteins (30 μg per lane) were resolved on a 10% (wt/vol) SDS-polyacrylamide gel (Novex, Groningen, The Netherlands) under reducing conditions. For immunoblotting, proteins transferred to a PVDF membrane. Blots incubated for 1 h. at 4 °C in a blocking buffer containing 5% skim milk. Later on, the mixture was incubated with either mouse anti- human\mice α-SMA (Novusbio; NB600–531)*,* rabbit anti-collagen I (ab34710), rabbit anti-VDR (ab109234) and mouse anti-human\mice β-Actin (R&D System; 937,215**),** diluted 1/1000, overnight in 4 °C, and subsequently, with peroxidase-conjugated goat anti-mouse and Rabbit IgG (PARIS, Compiegne, France) diluted 1/5000, for 1.5 h at room temperature. Immuno-reactivity revealed by enhanced chemiluminescence using an ECL Kit (Abcam; ab133406)**.**

### Histological assessments of liver injury

The posterior one third of the liver was fixed in 10% formalin for 24 h and then paraffin-embedded in an automated tissue processor. Seven-millimeter liver sections were cut from each animal. Sections (15 mm) were then stained in 0.1% Sirius red F3B in saturated picric acid as well as Masson trichrome stain for connective tissue stain (both from Sigma). Sirius red staining assessed using the modified Histological Activity Index (HAI) criteria, incorporating semi quantitative assessment of periportal/periseptal interface hepatitis (0–4), confluent necrosis (0–6), focal lytic necrosis/apoptosis and focal inflammation (0–4), portal inflammation (0–4), and architectural changes/fibrosis and cirrhosis (0–6).

### Statistical analysis

The results were evaluated using the Student’s t-test, with statistical significance set at *P* < 0.05. Comparison between the mean values of different experiments was carried out. All data were presented as mean ± SE.

## Results

### 25-(OH) D3 reduced liver injury in acute while worsen fibrosis in chronic model of CCl_4_

**Fig. 1:**
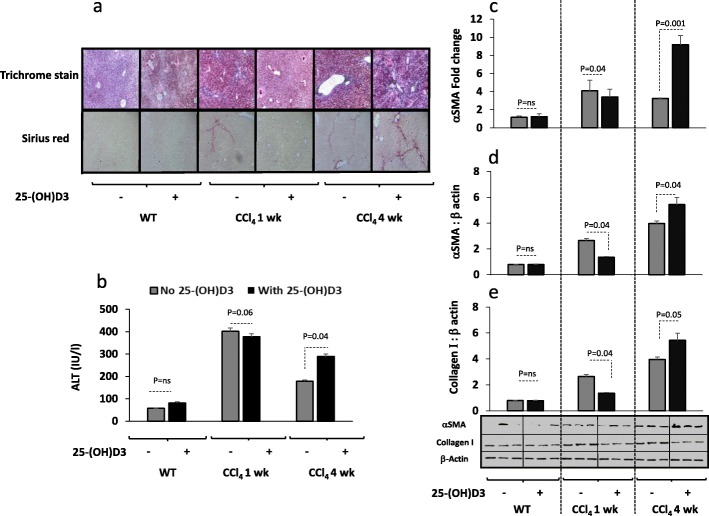
Vitamin D alleviates hepatic fibrosis in acute model of CCl_4_ while worsen in the chronic model. **a.** Liver histology assessments showed that vitamin D IP administration (0.5 mg/100 g body weight) caused a lack of hepatic fibrosis following staining with by the Trichrome and Sirius red in the 1 week injections of CCl_4_ (acute model) while aggravate collagen formations in the 4 weeks of CCl_4_ (chronic model). **b.** Serum ALT levels were significantly (*P* = 0.04) increased in the acute and chronic models of CCl_4_ and a further increased in ALT were seen following vitamin D treatments only in the chronic model (*p* = 0.04). Liver primary hepatic stellate cells (**c**.) RNA αSMA quantitation by RT PCR and western blot protein quantitation of (**d**.) αSMA and (**e.**) Collagen showed similar patterns to histology assessments

Hepatic fibrosis was induced in BALB/c mice by biweekly IP CCl_4_ injections for 1 week (acute model) and 4 weeks (chronic model) and was compared with naive vehicle-treated mice (WT) (*n* = 10 mice in each group). Histological staining and αSMA expressions were performed for fibrosis assessments. Figure 1a shows staining of Trichrome and Sirius red for the three major animal groups with and without IP administration of vitamin D as described in materials and methods. Acute and chronic model of CCl_4_ showed gradual extent of collagenous connective tissue fibers of red fibrosis septae with both Trichrome and Sirius red stains in consistent with CCl_4_ exposure time.

Fibrotic mice of WT and acute model of CCl_4_ treated with vitamin D showed attenuation in their fibrosis histology that lack red fibrosis septae. Vitamin D treatments in the chronic CCl_4_ model, in the contrary, had propagated liver fibrosis as seen in liver histological samples (Fig. 1a).

To evaluate effects of vitamin D on liver injury, systemic inflammatory marker for serum ALT levels and liver samples for quantitation of fibrosis were performed. Figure 1b shows serum ALT levels highly significantly (*P* = 0.05) as it elevated from 33.1 ± 7.5 IU/L in naïve WT mice to 401.5 ± 45 IU/L and 178 ± 21 IU/L in acute and chronic model of CCl_4,_ respectively. Vitamin D did not alter phenotype changes in WT mice, it inhibited systemic inflammation through decreased ALT levels in acute model of CCl_4_ to 380 ± 29.4 IU/L (*p* = 0.04) while propagate inflammation through increased ALT levels in the chronic model of CCl_4_ mice up to 288.5 ± 30.5 IU/L (*P* < 0.05). Gene expressions of liver αSMA fibrotic marker by RT PCR (Fig. 1c), αSMA liver portion expressions by western blot analysis (Fig. 1d) and liver collagen quantitation (Fig. 1e) showed similar patterns of ALT. The above results indicated dual anti- and pro- fibrotic effects of vitamin D that could be achieved depending on the severity of fibrosis.

### Liver VDR expressions were inhibited along fibrosis progressions and were unresponsive to vitamin D treatments

**Fig. 2:**
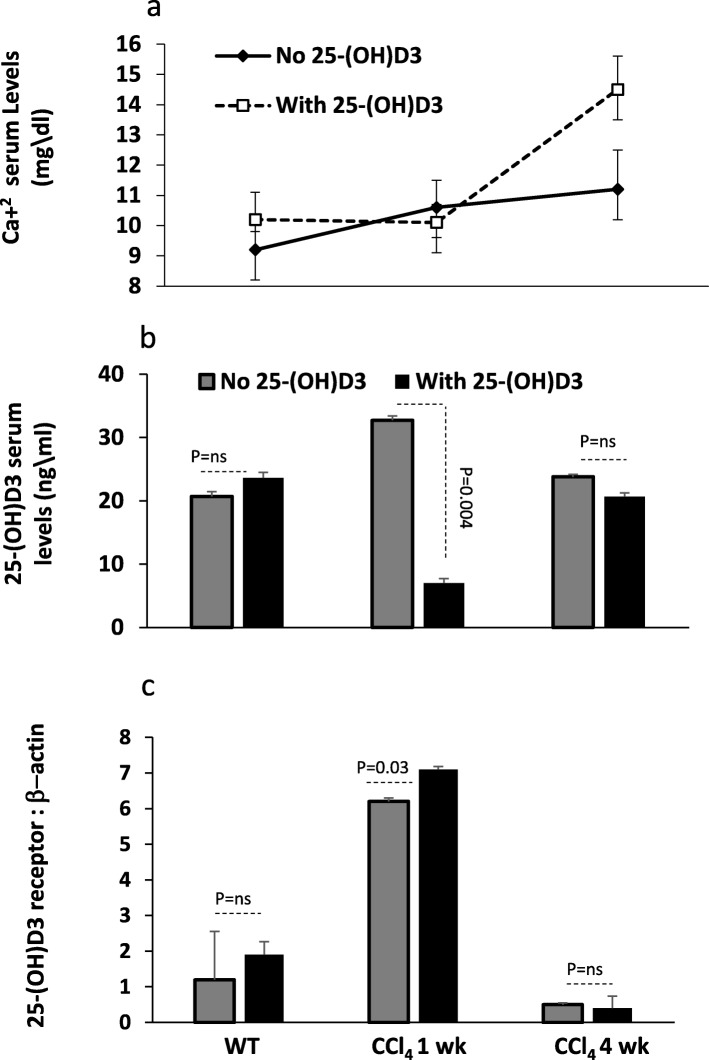
Chronic CCl_4_ mice model was associated with decreased pHSCs VDR and hypercalcemia. **a.** High Ca^+ 2^ serum levels were obtained following vitamin D treatments only on the chronic model (*p* < 0.05). **b.** A significant drop in serum vitamin D levels were seen in acute mice model of CCl_4_ following vitamin D treatments while no changes were seen in the chronic model. **c.** Inhibition of VDR in pHSCs in the chronic model of CCl_4_ while up-regulation of the VDR were obtained in acute model by western blot

The vitamin D hormone, 1,25-dihydroxyvitamin D(3) [1,25(OH)(2)D(3)], binds with high affinity to the nuclear vitamin D receptor (VDR), and is considered as primary regulator of calcium (Ca^+ 2^). We aimed to evaluate Ca^+ 2^, vitamin D and VDR changes in mice model treated with or without vitamin D. Prior to vitamin D treatments, similar Ca^+ 2^ serum levels were obtained in both the acute and the chronic CCl_4_- mice model (Fig. 2a; *P* = ns). Moreover, vitamin D treatments did not affect Ca^+ 2^ serum levels in both the WT and the acute model of CCl_4_, however, it significantly caused an elevation in Ca^+ 2^ levels as presented in the chronic model of CCl_4_.in Fig. 2a. We further display changes in mice phenotype and found vitamin D serum levels to be significantly elevated in the acute model of CCl_4_, an effect that were inhibited in the chronic model to levels similar to WT mice (Fig. 2b). Vitamin D treatments while did not alter vitamin D serum levels in the WT and chronic model of CCl_4_, it significantly dropped levels of serum vitamin D to 4.6 fold (*P* = 0.004) in the acute model.

Therefore, our next aim was to relate changes in serum vitamin with modulations in the VDR. For this purpose, primary hepatic stellate cells (pHSCs) obtained from livers from all mice groups were quantitated for VDR by the western blot analysis. Figure 2c shows upregulation in the expressions of VDR in acute model of CCl_4_ as compared to the WT, while total loss of VDR expressions were seen in the chronic model of CCl_4_ and indicate that VDR expressions were modulated along the course of the liver injury. Vitamin D treatments significantly caused increased in VDR expressions in acute model of CCl_4_ (*P* = 0.03) while showed to have no effects in VDR expressions in the WT and the chronic model of CCl_4_.

The above results suggest increased Ca^+ 2^ serum levels in chronic model of CCl_4_ treated with vitamin D could be a result of liver injury accompanied with vitamin D degradation. Consequently, these effects were associated with inhibited VDR and therefore, unlike the acute model, chronic mice model of CCl_4_ were unresponsive to vitamin D treatments (Fig. 2b and c). Moreover, due to the upregulation of VDR in the acute model of CCl_4_, vitamin D treatments were most probably up taken/ consumed and could explain the low vitamin D serum levels while calcium serum levels remained the same.

### NK cells have VDR and is crucial for their stimulations and modulation of liver fibrosis

**Fig. 3:**
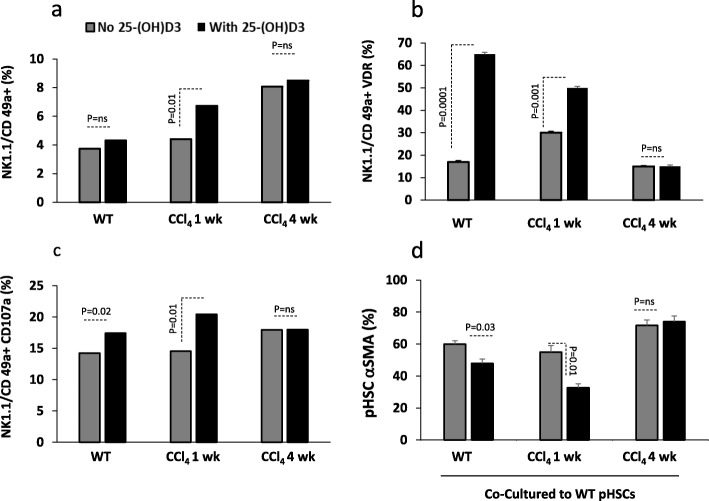
Increased NK 1.1/CD49a + in chronic model is correlated with their dysfunction and low VDR expressions. **a.** Liver NK 1.1/CD49a + cells showed gradual elevations in proliferations together with the extent of hepatic fibrosis; vitamin D treatments showed further liver NKs recruitments. **b.** NK 1.1/CD49a + were responsive to vitamin D treatments through increase in their VDR in the WT and acute CCl_4_ model however were unresponsive in the chronic model. **c.** NK 1.1/CD49a + showed similar CD107a in the vitamin D untreated mice with further stimulation in the WT and acute model of CCl_4_. **d.** NK1.1 cells from the WT and acute model of CCl_4_ showed to inhibit pHSCs αSMA percentages following co-culture assay following vitamin D treatments. NK1.1 cells from the chronic model of CCl_4_ showed no effects on αSMA percentages of pHSCs

We next sought to determine effects of vitamin D on immune alterations in liver fibrosis. We previously showed that NK cells exert anti-fibrotic effects through killing of activated HSCs [13]. We examined liver resident NK cells (NK1.1/CD49+) cellular infiltrates as well as their expressions of VDR and their activity (CD107a) by using the flow cytometry. Figure 3a shows a linear correlation between NK cells infiltrates with progression of liver fibrosis. Vitamin D caused an additional increase in the NK1.1 cells infiltrates (*p* = 0.01) only in the acute model of CCl_4_ indicating increased anti-fibrotic effects of NK1.1 cells and therefore inhibiting fibrosis in this model, a phenomena emphasized in our previous data summarized in Fig. 1 and Fig. 2. VDR expressions on liver NK1.1 increased in the acute while inhibited in the chronic model of CCl_4_ to levels similar to WT. The VDR were upregulated following treatments with vitamin D in the WT and acute model of CCl_4_. No changes in the VDR were noticed in the NK1.1 cell from chronic model of CCl_4_ following the vitamin D treatments (Fig. 3b). These data suggest inverse relation between NK1.1 cell proliferation (Fig. 3a) and their VDR expressions (Fig. 3b) prior and following vitamin D treatments.

To further correlate whether changes in the VDR on NK1.1 cells are associated with their phenotype alterations; CD107a (activity marker) was evaluated by the flow cytometry as described in materials and methods. CD107a NK1.1 percentages were high in both CCl_4_ models to similar levels of the WT. Vitamin D treatments further elevated NK1.1 cells activity in the WT and the acute model of CCl_4_ (*P* < 0.05) while did not alter NK1.1 activities in the chronic model most probably due to low VDR in the chronic model of CCl_4_. In order to determine whether changes in CD107a and VDR on NK1.1 cells could modulate liver fibrosis; we co-cultured NK1.1 cells from mice livers from all groups with WT pHSCs through an in vitro setting assay to assess cytotoxicity/killing potential. Figure 3d shows NK1.1 cells obtained from the acute model of CCl_4_ reduced αSMA percentages of pHSCs as compared to their WT counterparts. NK1.1 cells from the chronic model of CCl_4_ did not change αSMA of the pHSCs indicating inability of these cells to reduce fibrosis. To cite, HSCs monocultures staining revealed 90% αSMA (data not shown). Vitamin D treatments further exacerbate NK1.1 killing potentials in both the WT and the acute model and inhibited fibrosis while did not cause any changes in αSMA of the pHSCs in the chronic model of CCl_4_.

## Discussion

The cell-specific expression pattern of VDR suggests that the liver could be responsive to vitamin D during liver fibrosis through its non-parenchymal cells, in particular, HSCs. However, this was true only in the acute model of CCl_4_ for the following reasons: (1) VDR on HSCs was upregulated in the inflammatory model in contrary to the chronic (fibrotic) model. (2) Liver histology structure of collagen and αSMA is less pronounced in the inflammatory model and therefore vitamin D is less likely to undergo degradation. (3) Serum calcium levels were within the normal physiological range and is not expected to cause dramatic decrease in VDR as resulted in the fibrotic model.

Abramovitch and colleagues showed, in 2011, that ligation of VDR in HSCs inhibited their proliferation, and activation and reduced thioacetamide (TAA)-induced liver fibrosis in rats [14]. Further, Ding and colleagues revealed that VDR ligation in activated hepatic stellate cells has anti-fibrotic effects, which are mediated through a VDR/SMAD3/TGF-β signaling loop [15]. The authors also carried out genetic studies in mice which resulted in spontaneous liver fibrosis when one or both *Vdr* alleles were knocked out, with more severe fibrosis occurred in *Vdr*^*−/−*^ animals [15]. In our chronic fibrotic model, serum vitamin D levels was unchanged as compared to WT mice and progressions of liver fibrosis were due to inhibitions of VDR in HSCs. Also, vitamin D serum levels were unchanged following vitamin D treatments (Fig. 2b), in contrary to other studies that suggested that not only may vitamin D deficiency contribute to liver fibrosis [9].

Furthermore, the acute inflammatory model of CCl_4_ showed low serum vitamin D levels following vitamin D treatments a result that indicate consumption of vitamin D because of the high expressions of VDR. Therefore, low vitamin D serum levels in this case may not reflect liver injury while in the contrary showed alleviation in the fibrotic and the inflammatory profile. Moreover, our results showed modulation of VDR in liver resident NK cells of the chronic CCl_4_ model. NK cells of the fibrotic model showed less expressions of VDR and was correlated with their functional alterations. VDR inhibitions in NK cells from the chronic model of CCl_4_ attenuated their potentials to kill HSCs in our in vitro co-culture assay.

These findings, taken together with the correlation between liver VDR expressions and the severity of liver fibrosis, suggest that not only activated HSCs through their secretion of αSMA and collagen may contribute to liver fibrosis, it is also suggested that NK cells also lose their anti-fibrotic potential to kill HSCs, which exacerbate fibrosis especially when treated with vitamin D.

## Conclusion

Vitamin D showed to have dual effects on liver injury. In inflammatory acute model of CCl_4_, vitamin D reduced inflammatory and fibrotic markers while in the fibrotic chronic model of CCl_4_, vitamin D worsen fibrosis. These findings were associated with changes in liver NK cells phenotype that were seen to lose their activity and inability to kill activated HSCs in an in vitro settings. These results suggest that VDR changes is crucial for NK cell stimulations and consequently modulate liver fibrosis progressions. More studies are needed to clarify the role of VDR in attenuation of liver fibrosis.

## Data Availability

Data is available from the corresponding author upon reasonable request.

## Abbreviations

VDR: Vitamin D receptor
HSCs: Hepatic stellate cell
CCl_4_: Carbon tetrachloride
Vitamin D: 25-(OH) D3
